# Haplotypes of the *TaGS5-A1* Gene Are Associated with Thousand-Kernel Weight in Chinese Bread Wheat

**DOI:** 10.3389/fpls.2016.00783

**Published:** 2016-06-03

**Authors:** Shasha Wang, Xuefang Yan, Yongyan Wang, Hongmei Liu, Dangqun Cui, Feng Chen

**Affiliations:** National Key Laboratory of Wheat and Corn Crop Science, Collaborative Innovation Center of Henan Grain Crops, Agronomy College, Henan Agricultural UniversityZhengzhou, China

**Keywords:** bread wheat, *TaGS5-A1* gene, yield-related traits, thousand-kernel weight, single nucleotide polymorphism

## Abstract

In previous work, we cloned *TaGS5* gene and found the association of *TaGS5-A1* alleles with agronomic traits. In this study, the promoter sequence of the *TaGS5-A1* gene was isolated from bread wheat. Sequencing results revealed that a G insertion was found in position -1925 bp of the *TaGS5-A1* gene (Reference to ATG), which occurred in the *Sp1* domain of the promoter sequence. Combined with previous single nucleotide polymorphism (SNP) in the *TaGS5-A1* exon sequence, four genotypes were formed at the *TaGS5-A1* locus and were designated as *TaGS5-A1a-a, TaGS5-A1a-b, TaGS5-A1b-a*, and *TaGS5-A1b-b*, respectively. Analysis of the association of *TaGS5-A1* alleles with agronomic traits indicated that cultivars with the *TaGS5-A1a-b* allele possessed significantly higher thousand-kernel weight (TKW) and lower plant height than cultivars with the *TaGS5-A1a-a* allele, and cultivars with the *TaGS5-A1b-b* allele showed higher TKW than cultivars with the *TaGS5-A1b-a* allele. The differences of these traits between the *TaGS5-A1a-a* and *TaGS5-A1a-b* alleles were larger than those of the *TaGS5-A1b-a* and *TaGS5-A1b-b* alleles, suggesting that the -1925G insertion plays the more important role in *TaGS5-A1a* genotypes than in *TaGS5-A1b* genotypes. qRT-PCR indicated that *TaGS5-A1b-b* possessed the significantly highest expression level among four *TaGS5-A1* haplotypes in mature seeds and further showed a significantly higher expression level than *TaGS5-A1b-a* at five different developmental stages of the seeds, suggesting that high expression of *TaGS5-A1* was positively associated with high TKW in bread wheat. This study could provide a relatively superior genotype in view of TKW in wheat breeding programs and could also provide important information for dissection of the regulatory mechanism of the yield-related traits.

## Introduction

Wheat is one of the most important food crops in the world. However, with the rapid increase in world population, wheat production has been unable to meet the rate of population growth. Therefore, the improvement of wheat yield is crucial for resolution of the food crisis (Bhalla, 2006). Wheat yield is mainly controlled by genetic and environmental factors. To date, numerous yield-related quantitative trail loci (QTLs) have been mapped in bread wheat in the past decades, e.g., QTLs for plant height, spike length, kernel number per spike, kernel weight per spike, kernel length, kernel width, and thousand-kernel weight (TKW; Wang et al., 2009; Zhang et al., 2010; Cui et al., 2014; Liu et al., 2014; Bellucci et al., 2015; Xie et al., 2015).

However, it was very difficult to directly clone yield-related genes in hexaploid wheat so far due to its large genome size (≈ 17.9 Gb; Varshney et al., 2006). Since comparative genomics verified genomic collinearity between wheat and other crops (Gale and Devos, 1998), a few yield-related genes were gradually isolated from bread wheat by *in silico* cloning, e.g., sucrose synthase 2 orthologous gene (*TaSus2*; Jiang et al., 2011), putative cytokinin oxidase genes (*TaCKX2.1* and *TaCKX2.2*; Zhang et al., 2011), cell wall invertase gene (*TaCwi-A1* and *TaCwi-4A;* Ma et al., 2010; Jiang et al., 2015), *TaGW2* gene (Su et al., 2011; Hong et al., 2014), *1-FEH w3* (fructan exohydrolases) variant, *1-FEH-6B* (Van Riet et al., 2008; Zhang et al., 2009, 2015), and *TEF-7A* (Zheng et al., 2014). Of the yield-related genes, it has been proven that the *TaGW2* gene was significantly associated with TKW, heading, and maturity in bread wheat and that it negatively regulated kernel weight by controlling the gene expression level during seed development (Su et al., 2011; Bennarek et al., 2012; Hong et al., 2014; Qin et al., 2014; Simmonds et al., 2014).

To further understand gene function in bread wheat, the polymorphisms of many yield-related genes were examined, and corresponding molecular markers were developed for further utilization of marker-assisted breeding in bread wheat (Chono et al., 2015; Jiang et al., 2015; Zhang et al., 2015). A single nucleotide polymorphism (SNP) of the *1-FEH w3* gene in the -279 bp site of its promoter region was identified to be intimately associated with thousand-kernel weight (TKW) under drought conditions (Zhang et al., 2015). *TaGW2-6A, TaGW2-6B*, and *TaGW2-6D* genes were subsequently isolated from bread wheat. At the *TaGW2-6A* locus, wheat cultivars with *Hap-6A-A* were significantly associated with wider kernel and higher TKW (Su et al., 2011). At the *TaGW2-6B* locus, 11 SNPs in the promoter region formed four haplotypes (*Hap-6B-1, Hap-6B-2, Hap-6B-3*, and *Hap-6B-4*). Association analysis indicated that the *TaGW2-6B* has a stronger influence on TKW than *TaGW2-6A*, and *Hap-6B-1* was a relatively favored haplotype in view of grain width and weight (Qin et al., 2014).

More recently, a yield-related gene, *TaGS5-A1*, was reported to be associated with several agronomic traits including kernel size, TKW, and plant height in two previous studies (Wang et al., 2015; Ma et al., 2016). Two alleles, *TaGS5-A1a* and *TaGS5-A1b*, were found in Chinese bread wheat. Furthermore, wheat cultivars with *TaGS5-A1b* genotypes were associated with lower plant height and higher TKW in Chinese landraces and current cultivars, suggesting that *TaGS5-A1b* (named as *TaGS5-3A-T* by Ma et al., 2016) was a relatively preferable genotype and exhibited a larger potential application in wheat high-yield breeding.

Based on our previous study on *TaGS5* genes in bread wheat, we further analyzed the promoter sequence of the *TaGS5-A1* gene in different Chinese bread wheat cultivars in this study and discovered a new polymorphism of the *TaGS5-A1* gene as well as a relatively superior genotype in view of TKW of bread wheat. This study provides useful information for selection of a relatively preferable genotype in view of TKW in wheat breeding programs as well as provides important information for dissection of the regulatory mechanism of the yield-related traits of bread wheat.

## Results

### Cloning of the promoter of *TaGS5-A1* gene and discovery of an SNP insertion in bread wheat

**Figure 1:**
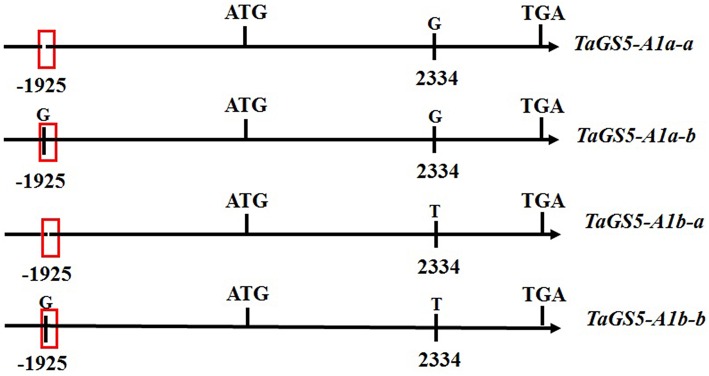
**Four genotypes and predicted ***Sp1*** domain of ***cis-acting*** regulatory elements in the promote region of ***TaGS5-A1*** gene**.

Based on the scaffold 21314 from URGI, a full-length 2287-bp promoter sequence of the *TaGS5-A1* gene was successfully cloned from bread wheat. Because *TaGS5-A1b* was a relatively preferable genotype in previous studies (Wang et al., 2015; Ma et al., 2016), 40 wheat cultivars with the *TaGS5-A1b* allele, 20 of which relatively large kernel and 20 relatively small kernel, were selected to sequence their promoter sequence of the *TaGS5-A1* gene. Finally, a nucleotide G insertion was found at position -1925 bp of the promoter of the *TaGS5-A1* gene (Figure S1 and Figure 1) (designated -1925G; BankIt 1916849). Sequencing results confirmed the reliability of this SNP. Furthermore, the prediction of a *cis*-acting regulatory element of the promoter sequence of *TaGS5-A1* gene found that the -1925G insertion was located in the *Sp1* domain (light responsive element) using Plantcare (http://bioinformatics.psb.ugent.be/webtools/plantcare/html/; Figure 1). Combined with G-T substitution in previous studies (Wang et al., 2015; Ma et al., 2016) and the -1925G insertion, four genotypes were formed and designated as *TaGS5-A1a-a* (no -1925G insertion and 2334G), *TaGS5-A1a-b* (-1925G and 2334G), *TaGS5-A1b-a* (no -1925G insertion and 2334T), and *TaGS5-A1b-b* (-1925G and 2334T) according to the nomenclature of McIntosh et al. (2007) (Figure 1).

### Association of the -1925G insertion in the promoter region of *TaGS5-A1* gene with agronomic traits

Based on PCR amplification with primer set TaGS5_−_P1 and sequencing results, 27 and 50 out of 77 *TaGS5-A1a* cultivars belonged to the *TaGS5-A1a-a* and *TaGS5-A1a-b* alleles, and 263 and 12 out of 275 cultivars belonged to the *TaGS5-A1b-a* and *TaGS5-A1b-b* alleles, respectively. Variance analysis at the *TaGS5-A1a* locus indicated that the plant height of cultivars with *TaGS5-A1a-a* alleles was significantly higher than those of cultivars with *TaGS5-A1a-b* alleles over 3 years. It also showed that panicle length of two genotypes was significantly different in 2014 yet not different in 2013 and 2015 and that internode length below the spike of two genotypes was significantly different in 2013 and 2014 yet not different in 2015 in the Chinese bread wheat cultivars surveyed (Table 1). Moreover, during the 3-year study, wheat cultivars with the *TaGS5-A1a-b* allele were found to possess significantly higher TKW than those cultivars with the *TaGS5-A1a-a* allele. Additionally, kernel length and kernel width of two genotypes were significantly different in 2014.

**Table 1:** **Comparison of agronomic traits of bread cultivars with ***TaGS5-A1a-a, TaGS5-A1a-b, TaGS5-A1b-a***, and ***TaGS5-A1b-b*** alleles**.

**Year**	**Trait/genotype**	***TaGS5-A1a-a***	***TaGS5-A1a-b***	***TaGS5-A1b-a***	***TaGS5-A1b-b***
	Sample no.	27	50	263	12
	Plant height (cm)	89.9 ± 4.76a	75.8 ± 2.01b	71.5 ± 1.02c	73.4 ± 3.08bc
	Panicle length (cm)	10.8 ± 0.42a	10.0 ± 0.21ab	10.3 ± 0.09ab	9.8 ± 0.45b
	Internode length below spike (cm)	30.2 ± 1.34a	25.3 ± 0.76b	25.0 ± 0.33b	24.9 ± 1.69b
	Spikelet number per spike	19.2 ± 0.39b	18.7 ± 0.27b	19.3 ± 0.14a	19.6 ± 0.63a
2013	Kernel number per spike	45.4 ± 3.44a	43.7 ± 1.68ab	42.8 ± 0.62b	44.6 ± 1.95ab
	Kernel length (mm)	6.80 ± 0.13a	6.97 ± 0.06a	6.85 ± 0.03a	6.88 ± 0.10a
	Kernel width (mm)	3.20 ± 0.05a	3.24 ± 0.03a	3.29 ± 0.01a	3.33 ± 0.04a
	Kernel length/kernel width ratio	2.13 ± 0.04a	2.16 ± 0.03a	2.0 ± 0.019a	2.08 ± 0.04a
	Thousand-kernel weight(g)	39.5 ± 1.67c	42.1 ± 1.01b	43.0 ± 0.43b	46.7 ± 1.54a
	Sample no.	27	50	263	12
	Plant height (cm)	99.8 ± 5.27a	78.5 ± 2.48b	77.6 ± 1.03b	80.1 ± 5.65b
	Panicle length (cm)	11.6 ± 0.58a	9.4 ± 0.24b	9.1 ± 0.10b	9.4 ± 0.47b
	Internode length below spike (cm)	35.0 ± 1.67a	29.2 ± 1.00b	29.8 ± 0.95b	29.0 ± 2.44b
	Spikelet number per spike	19.14 ± 0.36a	19.1 ± 0.26a	19.9 ± 0.11a	19.8 ± 0.40a
2014	Kernel number per spike	51.0 ± 1.76bc	47.8 ± 0.26c	52.94 ± 0.68ab	56.7 ± 2.31a
	Kernel length (mm)	6.83 ± 0.12b	7.03 ± 0.07a	6.93 ± 0.03ab	7.05 ± 0.12a
	Kernel width (mm)	3.32 ± 0.07b	3.55 ± 0.04a	3.73 ± 0.13a	3.65 ± 0.10a
	Kernel length/kernel width ratio	2.07 ± 0.05a	2.01 ± 0.03ab	1.92 ± 0.01b	1.94 ± 0.10b
	Thousand-kernel weight(g)	45.6 ± 1.87c	51.4 ± 1.01ab	50.7 ± 0.37b	52.3 ± 1.64a
	Sample no.	27	50	263	12
	Plant height (cm)	108.6 ± 4.35a	85.1 ± 1.95c	92.0 ± 1.95b	95.1 ± 4.39b
	Panicle length (cm)	11.5 ± 0.39a	10.7 ± 0.25b	10.4 ± 0.10b	10.5 ± 0.33b
	Internode length below spike (cm)	35.0 ± 1.24a	31.3 ± 1.78ab	30.3 ± 0.38b	30.5 ± 1.78b
	Spikelet number per spike	19.5 ± 0.51a	19.0 ± 0.33a	19.3 ± 0.12a	18.8 ± 0.51a
2015	Kernel number per spike	47.3 ± 2.13a	47.8 ± 1.41a	48.1 ± 1.41a	48.2 ± 2.38a
	Kernel length (mm)	6.79 ± 0.10a	7.03 ± 0.07a	6.82 ± 0.03a	6.88 ± 0.10a
	Kernel width (mm)	3.32 ± 0.04a	3.41 ± 0.04a	3.42 ± 0.01a	3.33 ± 0.07a
	Kernel length/kernel width ratio	2.05 ± 0.03a	2.07 ± 0.03a	2.00 ± 0.01a	2.08 ± 0.05a
	Thousand-kernel weight(g)	37.1 ± 1.51c	43.4 ± 1.22b	42.34 ± 0.46b	46.71 ± 3.04a

*Letters after numbers showed significant (P < 0.05) differences*.

Variation analysis at the *TaGS5-A1b* locus showed that plant height, panicle length, internode length below spike, spikelet number per spike, kernel number per spike, kernel length and kernel width, KL/KW ratio did not show any significant difference between cultivars with *TaGS5-A1b-a* and *TaGS5-A1b-b* alleles. However, cultivars with *TaGS5-A1b-b* possessed a significantly higher TKW than cultivars with *TaGS5-A1b-a* for this study of over 3 years of Chinese wheat cultivars. Furthermore, cultivars with *TaGS5-A1b-b* possessed the significantly highest TKW among cultivars with four *TaGS5-A1* alleles.

Therefore, the -1925G insertion contributed to a higher TKW at both *TaGS5-A1a* and *TaGS5-A1b* loci in the Chinese bread wheat cultivars surveyed. Further analysis indicated that the difference of the agronomic traits between cultivars with and without the -1925G insertion sharply reduced at the *TaGS5-A1b* locus when compared with the *TaGS5-A1a* locus. This finding suggests that the -1925G insertion played the more important role in modulating the TKW of cultivars with *TaGS5-A1a* than in cultivars with *TaGS5-A1b*. It could be that the *TaGS5-A1* gene has played the more important role in cultivars with the *TaGS5-A1b* allele than in cultivars with the *TaGS5-A1a* allele according to previous studies (Wang et al., 2015; Ma et al., 2016). Looking at the combined results of previous studies by Ma et al. (2016), and Wang et al. (2015), *TaGS5-A1b-b* was a relatively preferable genotype in view of TKW in bread wheat and could be considered for improvement of TKW in the Chinese wheat breeding program (Table 1). However, TKW showed significantly negative correlation with kernel number per spike (Table 2), suggesting that *TaGS5-A1b-b* genotype might be negatively associated with kernel number per spike in the Chinese bread wheat cultivars surveyed.

**Table 2:** **Correlation analysis of agronomic traits in the Chinese bread wheat cultivars**.

**Traits**	**Plant height (cm)**	**Panicle length (cm)**	**Internode length below spike (cm)**	**Spikelet number per spike**	**Kernel number per spike**	**Kernel length (mm)**	**Kernel width (mm)**	**Kernel length/kernel width ratio**
Panicle length (cm)	0.25^**^							
Internode length below spike	0.65^**^	0.21^**^						
Spikelet number per spike	−0.13^**^	0.27^**^	−0.15^**^					
Kernel number per spike	−0.13^**^	0.22^**^	−0.13^**^	0.59^**^				
Kernel length (mm)	−0.08	0.23^**^	0.01	−0.05	−0.21^*^			
Kernel width (mm)	−0.12^*^	−0.05	−0.09	0.02	0.02	0.04		
KL/KW ratio	0.16^**^	0.26^**^	0.18^**^	−0.11^*^	−0.18^**^	0.66^**^	−0.36^**^	
Thousand-kernel weight(g)	−0.24^**^	−0.03	−0.13^**^	−0.08	−0.28^**^	0.43^**^	0.18^**^	−0.12^*^

** and * *after numbers showed extreme (P < 0.01) and significant (P < 0.05) differences, respectively*.

### Expression analysis of *TaGS5-A1b-a* and *TaGS5-A1b-b* genotypes

**Figure 2:**
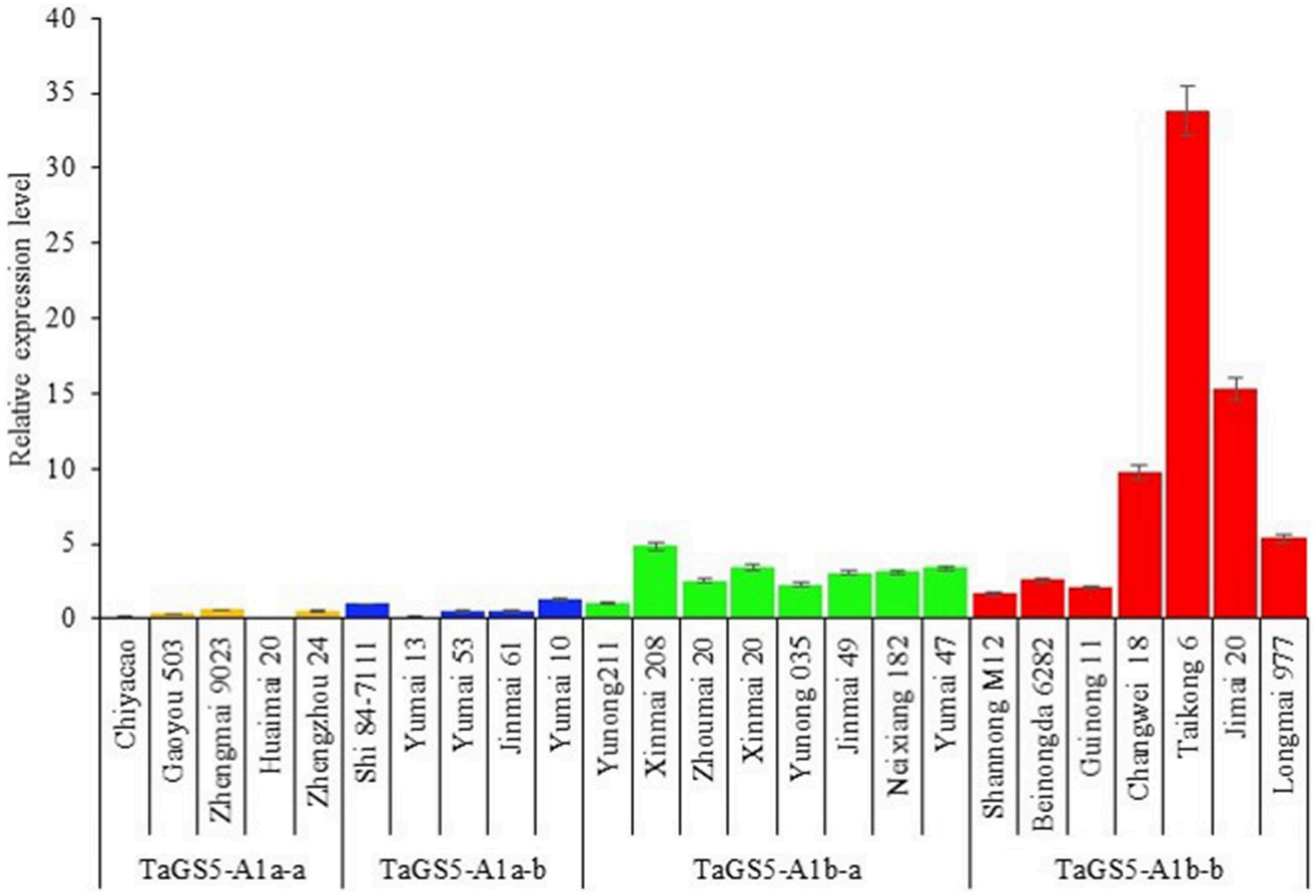
**Relative expression levels of 25 Chinese cultivars with different ***TaGS5-A1*** alleles in mature seeds**.

**Figure 3:**
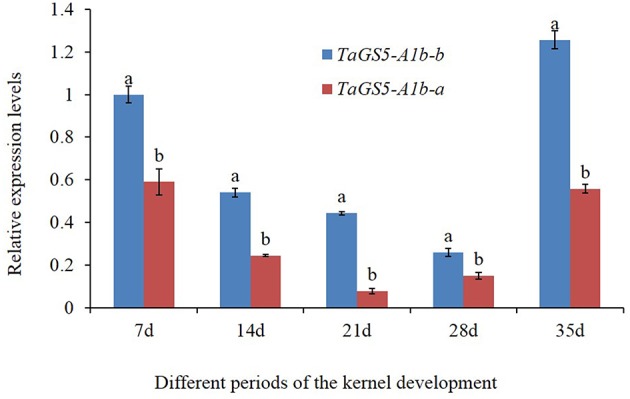
**Relative expression levels of ***TaGS5-A1b-a*** and ***TaGS5-A1b-b*** alleles in the seeds of different developmental stages**. Different letters on the top of the bars indicated the significant difference at 5% probability level.

The mature seeds of 25 Chinese cultivars (5 cultivars with *TaGS5-A1a-a*, 5 cultivars with *TaGS5-A1a-b*, 8 cultivars with *TaGS5-A1b-a* and 7 cultivars with *TaGSS5-A1b-b*) were used to analyze relative expression levels of *TaGS5-A1* gene. qRT-PCR results showed that relative expression levels of eight cultivars with *TaGS5-A1b-a* and seven cultivars with *TaGS5-A1b-b* were significantly higher than those of cultivars with *TaGS5-A1a-a* and *TaGS5-A1a-b* (Figure 2). Furthermore, 4 out of 7 cultivars with *TaGS5-A1b-b* showed significantly higher expression levels than those of cultivars with *TaGS5-A1b-a*. Furthermore, two cultivars Yunong 211 with *TaGS5-A1b-a* and Jimai 20 were selected to identify expression levels at five different developmental stages of the seeds (Figure 3). qRT-PCR results indicated that the relative expression level of *TaGS5-A1b-b* was significantly higher than that of *TaGS5-A1b-a* at all five of the stages (Figure 3), indicating that the -1925G insertion most likely improved the expression level of the *TaGS5-A1* gene.

## Discussion

The *TaGS5* gene has previously been cloned and was further identified to have at least two alleles, designated as *TaGS5-A1a* and *TaGS5-A1b*, at the *TaGS5-A1* locus, and the *TaGS5-A1b* genotype has been considered to be associated with a relatively lower plant height, wider kernel width, and higher KTW (Wang et al., 2015; Ma et al., 2016). In this study, a new polymorphism was discovered at the *TaGS5-A1* locus which was also intimately associated with kernel size and other agronomic traits. Furthermore, *TaGS5-A1a-b* and *TaGS5-A1b-b* genotypes showed significantly higher TKW at *TaGS5-A1a* and *TaGS5-A1b* loci, respectively, and *TaGS5-A1b-b* genotype showed the highest TKW among four *TaGS5-A1* genotypes. Based on the distribution of four *TaGS5-A1* genotypes in this study, the *TaGS5-A1b-b* genotype with the highest TKW showed the lowest percentage in the Chinese bread wheat cultivars. Therefore, *TaGS5-A1b-b* could be considered as a relatively preferred genotype for potential utilization in view of TKW in the current Chinese wheat breeding program. However, the increased TKW probably would cause the reduction of kernel number per spike due to their negative correlation in this study.

A previous study indicated that the *TaGS5-A1b* (*TaGS5-3A-T*) genotype has a higher enzymatic activity than that of the *TaGS5-A1a* (*TaGS5-3A-G*) genotype (Ma et al., 2016). *TaGS5-A1b* also exhibited a higher expression level than *TaGS5-A1a* at different stages of seed development (Wang et al., 2015). In this study, *TaGS5-A1a-b* genotype possessed significantly higher TKW and showed higher expression levels at *TaGS5-A1a* locus, and *TaGS5-A1b-b* genotype exhibited the highest TKW and the highest expression level in mature seeds among four haplotypes. Overexpression of the *TaGS5-A1b* genotype in transgenic rice showed larger kernel size and higher TKW than that of *TaGS5-A1a* (*TaGS5-3A-G*; Ma et al., 2016). Combining the results all studies thus far on *TaGS5-A1*, the expression level of *TaGS5-A1* has been positively associated with higher TKW in both wheat and rice. Therefore, overexpression or improving the enzymatic activity of the *TaGS5-A1* gene could possibly result in higher TKW and could be potentially used in a wheat breeding program.

At the *TaGS5-A1a* locus, *TaGS5-A1a-b* was a relatively preferred genotype when compared to *TaGS5-A1a-a* and possessed higher TKW and lower plant height, panicle length, and internode length below spike. Therefore, higher TKW and larger kernel size of cultivars with the *TaGS5-A1a-b* allele possibly resulted from its relatively lower plant height, shorter panicle length, and internode length below spike. However, further analysis indicated that the difference of the agronomic traits between cultivars with and without -1925G insertion sharply reduced at the *TaGS5-A1b* locus when compared with the *TaGS5-A1a* locus, and plant height, panicle length, and internode length below spike did not show a significant difference between cultivars with *TaGS5-A1b-a* and *TaGS5-A1b-b* alleles even though the *TaGS5-A1b-b* genotype still possessed a significantly higher TKW than the *TaGS5-A1b-a* genotype. The results suggested that the -1925G insertion in the *TaGS5-A1* gene played a more important role in modulating the TKW of cultivars with *TaGS5-A1a* than in cultivars with *TaGS5-A1b*. The reason was potentially because the TKW of bread wheat can also be modulated by other loci and cultivars with *TaGS5-A1b* had possessed a relatively higher expression at the *TaGS5-A1* locus and had showed a higher TKW. Therefore, it is possibly more effective for cultivars with *TaGS5-A1a* to select the -1925G insertion in the *TaGS5-A1* gene in view of TKW in a high-yield wheat breeding program. For cultivars with *TaGS5-A1b*, consideration of other yield-related loci was possibly more effective in improving TKW than the -1925G insertion in the *TaGS5-A1* gene. However, more work need to further understand the influence of the -1925G insertion on expression of the *TaGS5-1* gene as well as agronomic traits.

The polymorphism commonly occurred in the promoter region of yield-related genes in crops and thus resulted in diverse agronomic traits. For example, the *GS5* gene was firstly cloned in rice, and the polymorphism of the promoter region of the *OsGS5* gene caused variation of TKW by regulating kernel width and filling (Li et al., 2011). The *TaGW2* gene was cloned earlier in bread wheat by *in silico* cloning, and the polymorphism of its promoter region was identified to be intimately associated with TKW (Su et al., 2011; Qin et al., 2014; Simmonds et al., 2014). A yield-related gene, *TaCWI*, was identified as having four SNPs and two InDels in its promoter region and thus formed two haplotypes (*Hap-5D-G* and *Hap-5D-C*). *Hap-5D-C* genotypes showed lower plant height, earlier heading and maturity date, and higher TKW in Chinese modern wheat cultivars (Jiang et al., 2015). In this study, the -1925G insertion was discovered in the promoter region of the *TaGS5* gene and was intimately associated with high expression of the *TaGS5* gene as well as increased TKW, and it changed other agronomic traits. It was possible that the -1925G insertion might be able to activate expression of the *TaGS5-A1* gene and resulted in higher expression of the *TaGS5-A1* gene. More recently, two SNPs (*GS5-1* and *GS5-2*) in light-responsive elements of the promoter of *GS5* gene of rice were identified to be responsible for alteration of the response to light induction, leading to higher expression of *GS5-2* than *GS5-1* in leaves (Xu et al., 2015). Because the -1925G insertion was in the *Sp1* domain (light responsive element) of the *TaGS5-A1* gene, therefore, this study will also be useful for further analyzing the role of the *Sp1* domain in gene function as a *cis* element.

In this work, the promoter region of the *TaGS5-A1* gene was successfully cloned from the bread wheat, and a -1925G insertion was identified in this region. Further analysis indicated that the -1925G insertion was intimately associated with high TKW as well as plant height, spike length, and internode length below spike in Chinese bread wheat. In addition, the -1925G insertion played a greater role in the *TaGS5-A1a* genotype than in the *TaGS5-A1b* genotype. Furthermore, cultivars with the *TaGS5-A1b-b* allele, showing the highest expression at the *TaGS5-A1* locus in the mature seeds, exhibited relatively higher TKW. Therefore, this study provides a relatively preferable genotype at the *TaGS5-A1* locus in view of TKW for a wheat breeding program and could also provide useful information for understanding the molecular and genetics basis of yield-related traits in bread wheat.

## Materials and methods

### Plant materials and investigation of agronomic traits

In this study, a total of 352 wheat cultivars were used to analyze the effect of the -1925G insertion in the promoter region of *TaGS5-A1* gene on agronomic traits. These cultivars were mainly from the Yellow and Huai wheat regions of China, and were planted at the experimental field of Zhengzhou Scientific Research and Education Center of Henan Agricultural University (N34.9°, E113.6°) in 2012–2013, 2013–2014, and 2014–2015 cropping seasons. A randomized complete block design was adopted, and two replicates were performed. A specific experimental planting method was performed according to the method of Wang et al. (2015). A number of agronomic traits were investigated in the fields before harvesting, i.e., plant height, spike length, internode length below spike (ILBS), and spikelet number of ten spikes of each cultivar. After harvesting, kernel number of ten spikes, kernel length (KL), kernel width (KW), KL/KW ratio and thousand-kernel weight (TKW) of each cultivar surveyed were investigated using naturally dried seeds.

### DNA extraction, PCR amplification parameters, and sequencing

Genomic DNA was extracted from all wheat cultivars surveyed according to the method of Chen et al. (2010). DNA concentration and quality were detected using a Thermo Scientific NanoDrop 2000. PCR reaction system containing 25 μL. It mainly consisted of 0.5 μL primer sets (10 pmol/μl), 100 ng genomic DNA, 2.5 μL 10 × Taq buffer (Mg2+ plus), 0.5 μL dNTP (2.5 mM), and 1.25 U Taq DNA polymerases (Tiangen Biotech, Beijing Co. LTD.). PCR amplifications were performed using BioRad-S1000 or ABI 9700 thermal cyclers. The PCR reaction included three stages: 94°C for 5 min, 35 cycles (94°C for 30 s, 55–59°C annealing for 30 s and 72°C for 1–2 min), and 72°C for 7 min. PCR products were analyzed and separated on 1.0–1.5% (w/v) agarose gels, stained with ethidium bromide, and visualized with UV light.

The expected PCR products were purified using the SanPrep Column DNA Gel Extraction Kit (Shanghai Biological Technology Co., Ltd., Shanghai, China). The purified PCR products were ligated into pMD18-T vector (TaKaRa Biotechnology Co., Ltd., Dalian, China) and were transformed into cells of the Escherichia coli *DH-5*α strain. Plasmids containing targeted fragments were extracted by the Plasmid Rapid Isolation Kit (Shanghai Biological Technology Co., Ltd.) and 12 sub-clones for each sample were sequenced from both directions by Shanghai Sangon Biotech Co., Ltd. The sequenced results were analyzed using software DNAMAN Version 6.0 (http://www.lynnon.com/). The reliability of the sequencing results was checked by examining the sequence chromatograms using Chromas Version 1.4.5 and FinchTV 1.5.0 (http://www.geospiza.com/Products/finchtv.shtml).

### Primer design and identification of the SNP

Based on the URGI database (Unité de Recherche Génomique Info: https://urgi.versailles.inra.fr/) and the *TaGS5-A1* gene we cloned previously (Wang et al., 2015), a scaffold 21314 containing the *TaGS5-A1* gene was obtained from the URGI database. Based on the sequence of this scaffold, four pairs of primer sets (TaGS5_−_P1, TaGS5_−_P2, TaGS5_−_P3, TaGS5_−_P4 in Table 3) were designed to amplify the promoter sequence of the *TaGS5-A1* gene in Chinese Spring. Finally, a 2287-bp promoter sequence of the *TaGS5-A1* gene was successfully amplified in Chinese Spring. All primers (Table 3) in this study were designed by Premier Primer 3.0 software (http://primer3.ut.ee/) and Premier Primer 5.0 software, and were synthesized by Shanghai Biological Technology Co., Ltd. (http://www.sangon.com/).

**Table 3:** **All primers used for cloning the promoter sequence of ***TaGS5*** gene and qRT-PCR**.

**Primer**	**Primer sequence(5′-3′)**	**Annealing Temp (°C)**	**Size of PCR fragment (bp)**
TaGS5_−_P1	Forward:TCATACACACATAATCCAGTCGA Reverse:GATCGTGGGTGTTGCATCTAT	55	800
TaGS5_−_P2	Forward:GACTTAGAACCACGACAGCC Reverse:CGTAGCATCCATCGGCATG	57	1086
TaGS5_−_P3	Forward:GAGCACAAGAGTGAAGCGAGATGG Reverse:CGTTGTTGGCGTATGCGTCTGA	59	1400
TaGS5_−_P4	Forward:AAGGTCGGGCAAAGTCTATG Reverse:CGAGGAGAAAGAGAGCAAGGA	56	1000
18s	Forward:CCTGCGGCTTAATTGACTC Reverse: GTTAGCAGGCTGAGGTCTCG	56	150
TaGS5_−_P5	Forward:TAGAGCCTCAAACTGGACCG Reverse: AGATGCTGATGATGTTTGTCCA	56	127

### Quantitative RT-PCR of *TaGS5-A1b-a* and *TaGS5-A1b-b* alleles

Total RNA was extracted by a Trizol reagent and was reverse-transcripted to cDNA by a PrimeScript RT reagent kit with gDNA eraser (TaKaRa Biotechnology Co. Ltd, Dalian, China) per the kit instructions. Gene-specific primer set TaGS5_−_P5 were designed to examine the expression levels of *TaGS5-A1b-a* and *TaGS5-A1b-b* alleles. An *18s* gene was selected as the internal control with the primer 18s (Table 3). The mature seeds of 25 cultivars with different *TaGS5-A1* alleles (5 for *TaGS5-A1a-a*, 5 for *TaGS5-A1a-b*, 8 for *TaGS5-A1b-a*, and 7 for *TaGS5-A1b-b*) were used to analyze the relative expression levels of four *TaGS5-A1* alleles. Two wheat cultivars, Yunong 211 (193 days to flowering) with *TaGS5-A1b-a* allele and Jimai 20 (190 days to flowering) with *TaGS5-A1b-b* allele, were selected to compare expression levels of *TaGS5-A1b-a* and *TaGS5-A1b-b* alleles at the different stages of seed development. The immature seeds from Yunong 211 and Jimai 20 at the five stages (7, 14, 21, 28, and 35 d) after anthesis were sampled in the experimental field conducted at Zhengzhou Scientific Research and Education Center of Henan Agricultural University during the 2014-2015 cropping season for qRT-PCR. Quantitative RT-PCR was performed using the Bio-Rad iQ5 Sequence Detection System with the SYBR Premix Ex TaqII [TaKaRa Biotechnology Co., Ltd., Dalian, China]. The PCR conditions consisted of an initial denaturation step for 2 min at 94°C followed by 40 cycles of 10 s at 95°C, 10 s at 56°C, 20 s at 72°C, and a final extension of 10 min at 72°C. The *18s* gene was selected as the internal control. The 2^−ΔΔ*CT*^ method was used to normalize and calibrate transcript values relative to the endogenous *18s* control. Eight independent samples with triplicate repeats were analyzed.

### Statistical analysis

Coefficient of correlation among agronomic traits were performed by Excel 2013. Analysis of variance was conducted by PROC MIXED in the Statistical Analysis System (SAS Institute Inc., 2000) with genotype classes as categorical variables to derive the means of agronomic traits for each class and test the significant level for the four classes. The differences in agronomic traits among cultivars with different genotypes were tested by Tukey Honestly Significant Difference (HSD) using SPSS 19.0 for multiple comparisons.

## Author contributions

FC designed the project. SW and XY performed experiment. YW, HL, and DC investigated agronomic traits. FC and SW wrote the paper.

### Conflict of interest statement

The authors declare that the research was conducted in the absence of any commercial or financial relationships that could be construed as a potential conflict of interest.
