# Supplementary material for: Haplotypes of the TaGS5-A1 Gene Are Associated with Thousand-Kernel Weight in Chinese Bread Wheat
Source: Front Plant Sci. 2016 Jun 3;7:783. doi: 10.3389/fpls.2016.00783 (PMC4891348; doi:10.3389/fpls.2016.00783)
Supplement: Supplementary file 1 [file DataSheet1.DOCX]

*TaGS5-A1a-a*TCATACACACATAATCCAGTCGACCGCCTCCGGGCTCCGAGACATAGGGCTATTACTTCCTCCGAGAAGGGCCTGAACTC 80

*TaGS5-A1a-b* TCATACACACATAATCCAGTCGACCGCCTCCGGGCTCCGAGACATAGGGCTATTACTTCCTCCGAGAAGGGCCTGAACTC 80

*TaGS5-A1a-a* GTAAACCTTGTGTGCCTACAACTACTCCATAGCTAAGATCTTGCCTCTCCATACTACCCCCCTACCATTACTGTCAGACT 160

*TaGS5-A1a-b* GTAAACCTTGTGTGCCTACAACTACTCCATAGCTAAGATCTTGCCTCTCCATACTACCCCCCTACCATTACTGTCAGACT 160

*TaGS5-A1a-a* TAGAACCACGACAGCCGCCAGCAGCTGCCGCGCCCGCGCAGAGGGTTTTGACGGCGCCGTTGCTTACTCTCTGAACCGAT 240

*TaGS5-A1a-b* TAGAACCACGACAGCCGCCAGCAGCTGCCGCGCCCGCGCAGAGGGTTTTGACGGCGCCGTTGCTTACTCTCTGAACCGAT 240

*TaGS5-A1a-a* CGAACACAGTCGGGGATTTTGGGTGATCTCGCCGAAGCAAGTCACACCTGCGACGGTGGATGTTACCATTCTAGATCAAG 320

*TaGS5-A1a-b* CGAACACAGTCGGGGATTTTGGGTGATCTCGCCGAAGCAAGTCACACCTGCGACGGTGGATGTTACCATTCTAGATCAAG 320

*TaGS5-A1a-a* AGGATTGCAGAGCAACGTAGGATTTTTTTT.GGGGGGGGGGGCAAGAATCATTGGCTCTGATTACGAATTGTCACGCCCC 399

*TaGS5-A1a-b* AGGATTGCAGAGCAACGTAGGATTTTTTTTGGGGGGGGGGGGCAAGAATCATTGGCTCTGATTACGAATTGTCACGCCCC 400

*TaGS5-A1a-a* GATCTGGATCGTGGGCGACCAGATTAGGCTAGGGAAATCAGGAAGTGGATGAGCGAGACTTGGAGAAGGTGGTGAGCACA 479

*TaGS5-A1a-b* GATCTGGATCGTGGGCGACCAGATTAGGCTAGGGAAATCAGGAAGTGGATGAGCGAGACTTGGAGAAGGTGGTGAGCACA 480

*TaGS5-A1a-a* AGAGTGAAGCGAGATGGGATTTCGGAATTCATCAATATAGTCTGTCCACACACACCCTAATGGGCTTAAGTATAGATGCA 559

*TaGS5-A1a-b* AGAGTGAAGCGAGATGGGATTTCGGAATTCATCAATATAGTCTGTCCACACACACCCTAATGGGCTTAAGTATAGATGCA 560

*TaGS5-A1a-a* ACACCCACGATCAGCTGGCCACGCACGCACGAATCTCTGGTCCCTGGGACCCACTTGCCAGTCACACGGTGGTGTCGAGC 639

*TaGS5-A1a-b* ACACCCACGATCAGCTGGCCACGCACGCACGAATCTCTGGTCCCTGGGACCCACTTGCCAGTCACACGGTGGTGTCGAGC 640

*TaGS5-A1a-a* ACTGCCGGTGTGACAAAGTGCCAAAAGGATTTCAAGCCTGAATTATTTTTGCTCGGCCATACTATTAAGAGAAAGGCATA 719

*TaGS5-A1a-b* ACTGCCGGTGTGACAAAGTGCCAAAAGGATTTCAAGCCTGAATTATTTTTGCTCGGCCATACTATTAAGAGAAAGGCATA 720

*TaGS5-A1a-a* TTCCGAATTGCAATCTTGGATAGAAAGATTTGCATACTTCTCTCAATTTTCTTGCTTTTTGTATAACTGCCTTTGTAGAT 799

*TaGS5-A1a-b* TTCCGAATTGCAATCTTGGATAGAAAGATTTGCATACTTCTCTCAATTTTCTTGCTTTTTGTATAACTGCCTTTGTAGAT 800

*TaGS5-A1a-a* GTGCAAGCAAAGACAAACAAAAAAAATGCATGCAGTACAATTGTATGGGACGGTATGGGAAGTCCCCTCCATCAACAGAT 879

*TaGS5-A1a-b* GTGCAAGCAAAGACAAACAAAAAAAATGCATGCAGTACAATTGTATGGGACGGTATGGGAAGTCCCCTCCATCAACAGAT 880

*TaGS5-A1a-a* CGGGTCCGATACGGCTCAGCAGGTGACACATGTGTTTCTCCGGAGACGTCGAGCAATGCCTGTCGCCCTCAAGTGAAGCC 959

*TaGS5-A1a-b* CGGGTCCGATACGGCTCAGCAGGTGACACATGTGTTTCTCCGGAGACGTCGAGCAATGCCTGTCGCCCTCAAGTGAAGCC 960

*TaGS5-A1a-a* GTCGGCGATGCATGCCGATGGATGCTACGCACGGCGTCTCATGCGGAGGGGTCAAGTCATGCCTGCTACCGGCCGGCACG 1039

*TaGS5-A1a-b* GTCGGCGATGCATGCCGATGGATGCTACGCACGGCGTCTCATGCGGAGGGGTCAAGTCATGCCTGCTACCGGCCGGCACG 1040

*TaGS5-A1a-a* CACGGTGGCTCTGGCGGAGGTGTCATGTTTAGCCTGTTGCTGCTGGATCGAAGGTGGTGCGATAGTGGCAGGAGGCAGGC 1119

*TaGS5-A1a-b* CACGGTGGCTCTGGCGGAGGTGTCATGTTTAGCCTGTTGCTGCTGGATCGAAGGTGGTGCGATAGTGGCAGGAGGCAGGC 1120

*TaGS5-A1a-a* GGTATGGGATTTCTTTGTCTTCCGGTATCTTCTCCGGAGGTATCCGGTTATCGAGGAGTCGGTAGCCGGATATGGGATGT 1199

*TaGS5-A1a-b* GGTATGGGATTTCTTTGTCTTCCGGTATCTTCTCCGGAGGTATCCGGTTATCGAGGAGTCGGTAGCCGGATATGGGATGT 1200

*TaGS5-A1a-a* AAGACTTCAATGACGAGTTTGTGCACTCCGGTGGAAACACAAGATCTATGACCTGGCTATGTCGATGTGTGCCTGCGTCG 1279

*TaGS5-A1a-b* AAGACTTCAATGACGAGTTTGTGCACTCCGGTGGAAACACAAGATCTATGACCTGGCTATGTCGATGTGTGCCTGCGTCG 1280

*TaGS5-A1a-a* TGTCCTTACTGAAGATGGTGGATTGAAGCTTCACTTTAGGAATGAGAATACAGAGTTCAACCTTGGGTTGGACTCACCAA 1359

*TaGS5-A1a-b* TGTCCTTACTGAAGATGGTGGATTGAAGCTTCACTTTAGGAATGAGAATACAGAGTTCAACCTTGGGTTGGACTCACCAA 1360

*TaGS5-A1a-a* CATCGACGCACGTGTGGCAATTTCTTTTTGAAGGCGTAGCCTAGAAATTGTATATTTTTCATTTTAATTTTGTCTTATAT 1439

*TaGS5-A1a-b* CATCGACGCACGTGTGGCAATTTCTTTTTGAAGGCGTAGCCTAGAAATTGTATATTTTTCATTTTAATTTTGTCTTATAT 1440

*TaGS5-A1a-a* CATAGGGTTAGTTAGTGGCGGAGCCAACCTTTCAAAAAGGTCGGGCAAAGTCTATGCATGCATGGTGACGTTGCCAGTAT 1519

*TaGS5-A1a-b* CATAGGGTTAGTTAGTGGCGGAGCCAACCTTTCAAAAAGGTCGGGCAAAGTCTATGCATGCATGGTGACGTTGCCAGTAT 1520

*TaGS5-A1a-a* TTGCACCAGAGCATCAGCAATTAGCATTACTAACCATGTTCTGGTAATATTATTTAATAGAGACTGGCCGGAGCAGCCCG 1599

*TaGS5-A1a-b* TTGCACCAGAGCATCAGCAATTAGCATTACTAACCATGTTCTGGTAATATTATTTAATAGAGACTGGCCGGAGCAGCCCG 1600

*TaGS5-A1a-a* GGCAGCCGCCCCAGGTTGCCGGGAGGTAGGGTTAATGTCTATCAGATGGAGCTACTGTCGCGAGGCATTCAGCCTCGAAT 1679

*TaGS5-A1a-b* GGCAGCCGCCCCAGGTTGCCGGGAGGTAGGGTTAATGTCTATCAGATGGAGCTACTGTCGCGAGGCATTCAGCCTCGAAT 1680

*TaGS5-A1a-a* TTTAATTTTGCCTTTTTTTCGGGTCTGGTGTTCGGCTGCTGATTTCTCATTTTTAATAATATATGGCTACGCGCATCAAG 1759

*TaGS5-A1a-b* TTTAATTTTGCCTTTTTTTCGGGTCTGGTGTTCGGCTGCTGATTTCTCATTTTTAATAATATATGGCTACGCGCATCAAG 1760

*TaGS5-A1a-a* GCTTTCGAGAAAAAGAACATGTAGTACAATGGTACTGTTTTCGGTAAAAATGAAAAAGAAAGAGAGAAAACAGAAACTGC 1839

*TaGS5-A1a-b* GCTTTCGAGAAAAAGAACATGTAGTACAATGGTACTGTTTTCGGTAAAAATGAAAAAGAAAGAGAGAAAACAGAAACTGC 1840

*TaGS5-A1a-a* AAACGCCAATCTCACTTGTCTCATGCACGAAGGTTTCTACTGTATGCTCGTCCAACTTGATGAATCGGTACGTGTGACCG 1919

*TaGS5-A1a-b* AAACGCCAATCTCACTTGTCTCATGCACGAAGGTTTCTACTGTATGCTCGTCCAACTTGATGAATCGGTACGTGTGACCG 1920

*TaGS5-A1a-a* TGTCAGCGTTTAGTCAGTCACAAGGCAAGCCGCCAAAAGCATCAGACGCATACGCCAACAACGCACAGACACGAGTCGGA 1999

*TaGS5-A1a-b* TGTCAGCGTTTAGTCAGTCACAAGGCAAGCCGCCAAAAGCATCAGACGCATACGCCAACAACGCACAGACACGAGTCGGA 2000

*TaGS5-A1a-a* GATGCCCTCGGCAAGGCTGCGGTGCCGGTGCCGGTGCCGGTGCCGCGCAGCAAATAAAATTCGTTATCCCTGGTTCACAT 2079

*TaGS5-A1a-b* GATGCCCTCGGCAAGGCTGCGGTGCCGGTGCCGGTGCCGGTGCCGCGCAGCAAATAAAATTCGTTATCCCTGGTTCACAT 2080

*TaGS5-A1a-a* GCAGCCAAGCCGGCCAATGGCCTCCTCTCACTCTCACCCCCACCCCCACCCCCACCCCCCAGCTCTGAGCTCCCTCCAAC 2159

*TaGS5-A1a-b* GCAGCCAAGCCGGCCAATGGCCTCCTCTCACTCTCACCCCCACCCCCACCCCCACCCCCCAGCTCTGAGCTCCCTCCAAC 2160

*TaGS5-A1a-a* TTCACATGATCTTCTAGTGTGATCTCACTCTCACACAGATTGACTTGCGCCCAGCCCCTGCCTCTCTGCTCTTGCTGTCC 2239

*TaGS5-A1a-b* TTCACATGATCTTCTAGTGTGATCTCACTCTCACACAGATTGACTTGCGCCCAGCCCCTGCCTCTCTGCTCTTGCTGTCC 2240

*TaGS5-A1a-a* CAGCCAAGCCACTCACTCTCACATTTGCACATTCTCATG 2278

*TaGS5-A1a-b* CAGCCAAGCCACTCACTCTCACATTTGCACATTCTCATG 2279

Fig. S1 Full alignment of the promoter sequence of *TaGS5-A1a-a* and *TaGS5-A1a-b* alleles
